# Development, religious affiliation, and social context: Substance use disorders in Puerto Rican transitional age youth

**DOI:** 10.3389/fpsyt.2023.1076869

**Published:** 2023-02-16

**Authors:** David Saunders, Tamara Sussman, Thomas Corbeil, Glorisa Canino, Hector Bird, Margarita Alegria, Cristiane S. Duarte

**Affiliations:** ^1^Center for Intergenerational Psychiatry, Division of Child Psychiatry, New York State Psychiatric Institute/Columbia University Irving Medical Center, New York, NY, United States; ^2^Behavioral Sciences Research Institute, University of Puerto Rico School of Medicine, San Juan, PR, United States; ^3^Massachusetts General Hospital, Harvard Medical School, Boston, MA, United States

**Keywords:** religion, religiosity, religious denomination, transitional age youth, Puerto Rico, alcohol use disorder, tobacco use disorder, illicit substance use disorder

## Abstract

**Introduction:**

Transitional age youth (i. e., ages 16–24; TAY) use higher levels of substances than any age group in the United States. Understanding what factors increase substance use during TAY could suggest novel targets for prevention/intervention. Studies suggest that religious affiliation is inversely associated with substance use disorders (SUDs). However, the association of religious affiliation and SUD, including the role of gender and social context, has not been studied in TAY of Puerto Rican ethnicity.

**Methods:**

Using data from *N* = 2,004 TAY of Puerto Rican ethnicity across two social contexts—Puerto Rico (PR) and the South Bronx, NY (SBx)—we tested the association of religious identity (Catholic, Non-Catholic Christian, Other/Mixed, and no religious affiliation, or “None”), and four SUD outcomes (alcohol use disorder, tobacco use disorder, illicit SUD, and any SUD). Logistic regression models were used to examine the association between religious identity and SUDs, then we tested for interaction by social context and gender.

**Result:**

Half the sample identified as female; 30, 44, and 25% were 15–20, 21–24, and 25–29 years of age, respectively; 28% of the sample received public assistance. There was a statistically significant difference between sites for public assistance (22 and 33% in SBx/PR, respectively; *p* < 0.001); 29% of the sample endorsed None (38 and 21% in SBx/PR, respectively.) Compared to None, identifying as Catholic was associated with reduced risk of illicit SUD (OR = 0.51, *p* = 0.02), and identifying as Non-Catholic Christian with reduced risk for any SUD (OR = 0.68, *p* = 0.04). Additionally, in PR but not SBx, being Catholic or Non-Catholic Christian was protective for illicit SUD when compared to None (OR = 0.13 and 0.34, respectively). We found no evidence of an interaction between religious affiliation and gender.

**Discussion:**

The percentage of PR TAY who endorse no affiliation is higher than the general PR population, reflective of increasing religious non-affiliation among TAY across cultures. Critically, TAY with no religious affiliation are twice as likely as Catholics to have illicit SUD, and 1.5 times as likely as Non-Catholic Christians to have any SUD. Endorsing no affiliation is more adverse for illicit SUD in PR than the SBx, underscoring the importance of social context.

## Introduction

### Transitional age youth and substance use disorders

Transitional age youth (TAY; typically defined as 16–24) use higher levels of alcohol and other substances than any other age group in the United States (US) (1). Specifically, the 2020 National Survey on Drug Use and Health found that among young adults ages 18–25, 5.2 million (15.6%) had an alcohol use disorder, and 4.9 million (14.6%) had an illicit drug use disorder, including 4.5 million with cannabis use disorder. Critically, substance use among TAY leads to significant adverse outcomes, including increased morbidity and mortality, as well as a wide array of cognitive, social, emotional, and behavioral outcomes (2). Thus, improved understanding of what factors increase substance use risk during this uniquely vulnerable transitional period could suggest novel targets for prevention and intervention.

The TAY developmental stage is distinguished from adolescence and adulthood by a variety of factors that likely contribute to increased risk for substance use disorders (SUDs). For example, TAY assume progressively more independence as they shift from adolescence to adulthood. This increased freedom is related to important developmental tasks such as identity formation in several areas, which may include confirming or departing from their family's identification with specific religious groups. Young adulthood also means increasing exposure to situations that involve substance use (3). For these reasons, identification of protective factors for SUDs is paramount for the TAY developmental period.

### Religious affiliation and substance use disorders

One potential protective factor is affiliation with a religious tradition (hereafter, “religious affiliation” or “religious identity”) (4–6), with some religious traditions being more protective than others. Indeed, multiple studies have shown SUD outcomes relate to affiliation with particular Christian denominations (i.e., Catholics vs. Protestants) (5, 6), including in adolescents (7). For example, in a Swiss sample of young men, Roman Catholic individuals were less likely to display at-risk cannabis use than Protestants and other Christians, and less likely to use mushrooms than other Christians (6). A study of adults in the US found that affiliation with specific religious traditions was associated with multiple drinking-related outcomes, including abstinence vs. drinking and moderate vs. heavy use. As an example, in the 2000 National Alcohol Survey of adults residing in the US, identification as Mormon, Muslim, Church of God, Pentecostal, Protestant/miscellaneous denominations, or Baptist, was associated with abstinence, while being Jehovah's Witness, Lutheran, or Catholic was associated with drinking alcohol (5). Furthermore, among adolescents in the U.S., affiliation with a fundamentalist Protestant tradition (8) (in comparison to Baptist, mainline Protestant, Roman Catholic, and other/unaffiliated) was inversely associated with alcohol use disorder (AUD), cannabis use disorder, cocaine use disorder, and any illicit SUD (7).

The reasons for each of the above findings likely varies by substance, context, and religious tradition. One might argue that these associations could stem from prescriptive rules about the use of certain substances in some traditions (e.g., Mormons strictly forbid alcohol consumption), and/or the sense of belonging and community that can be part of one identifying with specific religious traditions.

### Risk and protective factors for substance use disorders among transitional age youth

TAY who are marginalized and excluded from access to resources may face additional challenges. For example, TAY who are marginalized may also experience poverty may rely on under-resourced programs like public assistance, which may not be able to meet their needs. Indeed, our work over the past 20 years with the Boricua Youth Study (BYS)—a longitudinal study of two subsamples of Puerto Rican youth from the South Bronx, NY, US (hereafter, SBx) and San Juan, Puerto Rico (hereafter, PR)—has described and characterized the adverse mental and physical health outcomes that can result from the complex and combinatory effects of such forces as marginalization and poverty on youth of Puerto Rican descent (9–12). Further, as Puerto Rican individuals are at particularly high risk for SUDs relative to the general US Latine population (13, 14), identification of protective factors might be especially critical for this population.

Religious affiliation may be uniquely protective among TAY in communities that strongly emphasize communal and religious norms, such as Puerto Rican TAY. However, it is also possible that identification with a specific group may generate conflict and distress and, in this case, pave the way to substance misuse (15–18). In any case, the relationship between a certain religious affiliation on SUDs has yet to be tested in Puerto Rican TAY.

Complicating matters, additional factors have been shown to impact the association between religious affiliation and SUDs among TAY (19). For example, social context may play a role. A recent study of Indonesian Muslim adolescents found that the religiosity of the social context—including peers, family, community, culture—predicted lower substance use outcomes more than an individual's religious practice or belief (19). The authors argue that the “deterrent effect of religiosity requires external support from the social environment and community to become significant”, underscoring the notion that social context is a critical factor in the association between religiosity and SUDs (19). Because the religiosity of the Puerto Rican social context can differ between the island of PR and the SBx in NYC (20, 21), one might argue that the protective effect of religiosity on SUDs would be stronger in the social context where religiosity is a more integral part of the social environment in which one's religious affiliation is embedded.

Religious affiliation likely also intersects with gender identity. Indeed, evidence suggests that gender interacts with indices of religiosity/spirituality to affect the development of SUDs (22, 23). Specifically, Debnam et al. found that spirituality may help male (but not female) adolescents in reacting to stress, and therefore buffer against substance use (22). In a study of adolescents from Nova Scotia, Canada, religious service attendance and religious importance were differentially protective based on gender (23). In this case, religious importance was found to be protective against cannabis use in males but not females; and religious attendance was found to be protective against only binge drinking in males, but all substances in females. Thus, one might argue that a similar interaction between gender and religious identification could be present among TAY of Puerto Rican ethnicity, with implications for SUDs.

### Objectives

To that end, we sought to study the association of religious affiliation and SUDs, including the interaction of social context and gender, among TAYs in the BYS—a sample that is balanced by site (the SBx and PR), and gender (9, 10). Indeed, our previous work with the BYS as well as several prior studies has demonstrated that rates of SUDs vary by site and gender, with TAYs in PR displaying lower rates of most SUDs compared to those in the SBx, and, congruent with prior studies, women displaying lower rates of SUDs than men (10). Additionally, we have found that rates of SUDs in the BYS vary based on empirically defined religiosity/spirituality profile (in preparation) (24), with highly religious (devout) participants displaying the lowest rates of SUDs, and those who were spiritual but not religious displaying the highest.

We aim to (1) characterize the religious identities of Puerto Rican TAY across two contexts; (2) test the association between religious affiliation and SUDs; and (3) test interactive effects of either social context or gender in the association between religious affiliation and SUDS. Regarding our second aim, we hypothesize Catholics will display the lowest rates of SUDs, and that individuals who identify with any specific religion (Catholic; Non-Catholic Christian; or Other/Mixed) will have lower rates of SUDs compared to those who do not endorse any religious affiliation or identity (also known as “Nones”, a term used in the religious studies literature that refers to those who check the “None” box when asked for their religious affiliation) (25). Regarding our third aim, we hypothesize the religious affiliation of the majority of the population in the island of PR (Catholic) will have a stronger protective effect for SUDs among island than US continental Puerto Rican TAY youth. Given the diverse set of findings in the literature, we do not have a specific hypothesis about how gender will interact with religious affiliation in relation to risk of SUDs.

## Methods

We utilized data from the aforementioned Boricua Youth Study. All procedures have been reviewed and approved by the New York State Psychiatric Institute and University of Puerto Rico Medical School Institutional Review Boards. The most recent wave of data collection (Wave 4, collected 2013–2017) included 2,004 participants ages 15–29 [mean (SD) 22.6 (2.9); 95.6% ages 18 or over] from the SBx and the island of PR.

TAY self-reported gender as a binary independent variable (Male/Female). Religious affiliation, also self-reported by TAY, was determined based on response to the question “With what religion, doctrine or beliefs do you identify?”, which had thirteen possible answer choices. Responses were then collapsed into four religious identities: “Catholic” (Roman Catholic and Charismatic Catholic); “Non-Catholic Christian” (Protestant, Disciples of Christ, Pentecostal, 7th Day Adventist, Jehovah's Witness, and Mita Congregation); and “Other/Mixed” (Jewish, Muslim, Mixed religious preferences, and Other religious preference); “None” (No religious affiliation/identity). A dichotomous variable contrasted the first three groups with Nones (Religiously affiliated vs. Nones).

Four DSM-IV substance use outcomes served as dependent binary variables (yes or no), as assessed by the World Mental Health Organization Composite International Diagnostic Interview (26): (1) Past Year Tobacco Dependence (hereafter, Tobacco Use Disorder; TUD); (2) Past Year Alcohol Abuse/Dependence (hereafter, AUD); (3) Lifetime Illicit Substance Abuse/Dependence (hereafter, illicit SUD); and (4) endorsement of TUD, AUD, or illicit SUD (hereafter, any SUD; no requirement for use of more than one substance).

Descriptive demographic information [religious affiliation, gender, age group (15–20, 21–24, 25–29 years of age) and receipt of public assistance status (yes or no)] were summarized for the entire sample and by study site at enrollment. Chi square tests were used to assess differences between each study variable by site, as well as differences in religious affiliation by gender, public assistance, and age group.

Using logistic regression models, we tested separate models examining the association of religious affiliation (four category variable) with the four SUDs, adjusting for recruitment context (SBx or PR), gender, age group, and public assistance status. These steps were then repeated, substituting a dichotomous religious affiliation variable (Religiously affiliated/None) for the four-category religious affiliation variable. Finally, we tested interaction terms in separate models for site and religious affiliation, as well as gender identity and religious affiliation, to determine whether associations between religious affiliation and SUDs were different between site and/or gender or categories.

## Results

Descriptive statistics by site for religious affiliation, gender, age, and public assistance are reported in Table 1. Site differences were observed for religious affiliation (*p* < 0.01, df = 3); a higher proportion of participants from the SBx reported no religious affiliation than in PR (37.7 vs. 21.0%), while the number of participants identifying as Non-Catholic Christian was higher in PR (43.5 vs. 22.2% in the SBx). Sites did not differ by gender (*p* = 0.97) or by age (*p* = 0.92). However, public assistance varied by site such that more participants reported public assistance in the SBx than in PR (*p* < 0.01). TAY gender, age and public assistance (Wave 4) were used as covariates in these analyses, while results were stratified by site.

**Table 1:** Descriptive statistics, total, and by site.

			**South Bronx (*****N*** = **902)**		**Puerto Rico (*****N*** = **1,051)**		
**Variable**	**Total** ***N***	**Weighted %**	***N***	**Weighted %**	***N***	**Weighted %**	*p* ^*^
**Religious affiliation**							**<0.001**
1. None	552	28.68	338	37.74	214	20.95	
2. Catholic	668	34.54	323	35.52	345	33.71	
3. Non-Catholic Christian	673	33.69	202	22.20	471	43.49	
4. Other/mixed	60	3.09	39	4.53	21	1.85	
**W4 gender**							0.97
1. Male	946	50.32	445	50.38	501	50.28	
2. Female	1,007	49.68	457	49.62	550	49.72	
**W4 age group**							0.92
1.15–20	546	30.34	252	30.92	294	29.84	
2.21–24	897	44.57	407	44.12	490	44.95	
3.25–29	510	25.10	243	24.96	267	25.21	
**W4 public assistance**							**<0.001**
1. No	1,400	71.74	694	77.17	706	67.11	
2. Yes	553	28.26	208	22.83	345	32.89	

^*^p-value corresponds to the design-adjusted Rao-Scott chi-square test. Bold indicates that the data are statistically significant.

In additional chi-square tests, religious affiliation also differed by gender (*p* < 0.001; Table 2), with 33.3% of males reporting None as their religious affiliation compared to only 24.0% of females. Religious affiliation also differed by public assistance use (*p* = 0.02; not shown), such that more participants who identified as Non-Catholic Christian used public assistance than other reported identities. Religious affiliation did not significantly differ by age (*p* = 0.88; not shown).

**Table 2:** Religious affiliation by gender.

	**Male (*****N*** = **946)**			**Female (*****N*** = **1,007)**			
	**Frequency**	**Weighted % of gender**	**Weighted % of religious affiliation**	**Frequency**	**Weighted % of gender**	**Weighted % of religious affiliation**	**p** ^*^
None	312	33.3	58.5	240	24.0	41.5	<0.001
Catholic	295	31.9	46.4	373	37.3	53.6	
Non-Catholic Christian	309	31.8	47.4	364	35.6	52.6	
Other/Mixed	30	3.0	49.4	30	3.1	50.6	
**Total**	946			1,007			

^*^p-value corresponds to the design-adjusted Rao-Scott chi-square test.

After adjusting for covariates, statistically significant associations between religious affiliation and substance use disorders were found (Table 3A). Consistent with our hypothesis regarding our second aim, that Catholics will display the lowest rates of SUDs, compared to no religious affiliation (None), identifying as Catholic was associated with reduced odds of illicit SUD (OR = 0.51, CI = 0.29–0.89, *p* = 0.02). Confirming our hypothesis that individuals who identify with a specific religion will have lower rates of SUDs compared to those who do not identify with any, we found that identifying as Non-Catholic Christian was associated with reduced risk for any SUD compared to None (OR = 0.68, CI = 0.47–0.99, *p* = 0.04), while identifying as Other/Mixed was associated with increased risk for any SUD (OR = 1.96, CI = 1.01–3.84, *p* = 0.05). There were no statistically significant associations between religious affiliation and past year TUD or past year AUD (Table 3).

**Table 3:** Religion and substance use^*^.

**(A) Religious affiliation**												
	**Alcohol**			**Tobacco**			**Illicit SUD**			**Any SUD**		
	**OR**	**CI**	***p***	**OR**	**CI**	***p***	**OR**	**CI**	***p***	**OR**	**CI**	***p***
None	Ref			Ref			Ref			Ref		
Catholic	1.02	(0.61–1.71)	0.94	0.67	(0.41–1.1)	0.11	0.51	(0.29–0.89)	**0.02**	0.76	(0.52–1.09)	0.14
Non-Catholic Christian	0.61	(0.35–1.05)	0.08	0.68	(0.41–1.12)	0.13	0.64	(0.35–1.2)	0.17	0.68	(0.47–0.99)	**0.04**
Other/Mixed	2.01	(0.84–4.8)	0.11	2.14	(0.95–4.81)	0.07	1.38	(0.46–4.13)	0.56	1.96	(1.01–3.84)	**0.05**
**(B) Any religious identity vs. no religious identity**												
	**Alcohol**			**Tobacco**			**Illicit SUD**			**Any SUD**		
	**OR**	**CI**	***p***	**OR**	**CI**	***p***	**OR**	**CI**	***p***	**OR**	**CI**	***p***
Any religious affiliation^∧^	0.88	(0.56–1.38)	0.57	0.75	(0.5–1.13)	0.17	0.62	(0.37–1.01)	0.05	0.78	(0.57–1.07)	0.12

^*^All analyses adjusted for age, site (SBx vs. PR), and welfare recipient status (yes/no); ^∧^reference group, no religious affiliation. Bold indicates that the data are statistically significant.

In adjusted analyses testing for relationships between religious affiliation vs. no religious affiliation with SUDs and gender, no statistically significant associations were identified (Table 3B).

Consistent with our hypothesis regarding our third aim, that religious affiliation of the majority of the population in the island of PR (Catholic) will have a stronger protective effect for SUDs among island than US continental Puerto Rican TAY youth, we found an interaction between site and religious affiliation for illicit SUDs (*p* < 0.001; Table 4; the Other/Mixed religious affiliation category was dropped because of full separation in the stratified model for PR, as no participants who identified as “Other/Mixed” also reported SUD in PR). In PR, being Catholic or Non-Catholic Christian was protective for illicit SUD when compared to None (OR = 0.13; CI = 0.04–0.37; and OR = 0.34, CI = 0.14–0.88, respectively). The same, however, was not true in the SBx (OR = 0.98; CI = 0.53–1.81; and OR = 0.98, CI = 0.47–2.03, respectively). We found no evidence of an interaction between site and religious affiliation for the other three SUDs. We found no evidence of an interaction between religious affiliation and gender for any of the SUDs (TUD: *p* = 0.24; AUD: *p* = 0.79; illicit SUD: *p* = 0.10; any SUD: *p* = 0.48).

**Table 4:** Interaction of religious affiliation with site and illicit substance use disorder^*^.

			**No (*****N*** = **803)**		**Yes (*****N*** = **60)**				
**South Bronx**	**Total** ***N***	**Weighted %**	***N***	**Weighted row %**	***N***	**Weighted row %**	**OR**	**CI**	***P***
Religious affiliation	863								
None	338	39.53	311	91.93	27	8.07	Ref		
Catholic	323	37.21	303	93.40	20	6.60	0.98	(0.53–1.81)	0.94
Non-Catholic Christian	202	23.26	189	93.00	13	7.00	0.98	(0.47–2.03)	0.96
			**No (*****N*** = **996)**		**Yes (*****N*** = **34)**				
**Puerto Rico**	**Total** ***N***	**Weighted %**	***N***	**Weighted row %**	***N***	**Weighted row %**	**OR**	**CI**	***p***
Religious affiliation	1,030								
None	214	21.35	199	90.81	15	9.19	ref		
Catholic	345	34.34	340	98.86	5	1.14	0.13	(0.04–0.37)	**<0.001**
Non-Catholic Christian	471	44.31	457	96.85	14	3.15	0.34	(0.14–0.88)	**0.03**

^*^All analyses adjusted for age, site (SBx vs. PR), and welfare recipient status (Yes/No); bold = statistically significant.

## Discussion

### Religious affiliation and substance use disorders in Puerto Rican transitional age youth

In the first population-based study investigating religious affiliation and SUDs among Puerto Rican TAY, several notable findings emerged. Consistent with our hypothesis that Catholic affiliation would be associated with the lowest rates of SUDs due to being the religion of the majority of the population, we found that identifying as Catholic was associated with reduced odds of illicit SUD, compared to other groups. Consistent with our prediction that individuals with a specific religion affiliation compared to those who are unaffiliated will have lower rates of SUDs, identifying as Non-Catholic Christian was associated with reduced risk for any past year SUD compared to participants with no affiliation, while identifying as Other/Mixed was associated with increased risk for any SUD. Furthermore, we found evidence supporting our hypothesis that the religious affiliation of the majority of the population in the island of Puerto Rico (Catholic) will have a greater protective effect for SUDs among island when compared to US continental Puerto Rican TAY youth. More specifically, we found an interaction between site and religious affiliation for illicit SUDs, such that in PR, but not in the SBx, being Catholic or Non-Catholic Christian was protective for illicit SUD when compared to None. We did not find evidence supporting an interaction between site and religious affiliation for the other three SUDs, nor evidence of an interaction between religious affiliation and gender for any of the SUDs.

### Demographic findings

The percentage of TAY who endorse no religious affiliation in both sites of the BYS sample differs significantly from the general Puerto Rican population (27). Specifically, based on a 2014 Pew Report, 20 and 8% of Puerto Rican adults living in the US and PR, respectively, do not identify with a religious tradition. In our sample of TAY, the same values are 38 and 21% of TAY (27) in the mainland US and PR, respectively. Interestingly, the majority of the participants in the present sample are Millennials (individuals born between 1981 and 1996), in whom similar increases in religious non-affiliation have been observed within other social and cultural settings, including the US (25, 28).

The increase in religious non-affiliation is noteworthy from a demographic perspective because it suggests that not identifying with religion may be a generation-wide phenomenon, and not specific to particular ethnic or cultural communities, including Puerto Rican communities in the mainland and on the island of PR. This finding can be explained in multiple ways. One might hypothesize that the increase in TAY who do not endorse a religious affiliation is unique to Millennials; or alternatively, that it reflects spiritual seeking and religious identity development that is characteristic of the TAY from a developmental perspective. Our data and results do not allow us to conclude one way or another, indicating that more research on religious affiliation among TAY is needed—including how Millennials compare to past (e.g., Gen X and Baby Boomers) and future generations, such as Generations Z (born between the late 1990s and 2010) and Alpha (born after 2010).

The demographic findings are also important because they suggest that religious affiliation has an evolving nature—namely, that it is a fluid and dynamic phenomenon responsive to and reflective of multiple variables, including age, generation, and social and geographic context. In this way, the data underscore that religious identity may not be static, but rather subject to such forces as time and place.

### Implications for clinicians and researchers

Given the association between religious affiliation and SUDs, this point is not merely an academic one. Indeed, the shifts in religious affiliation could have significant clinical ramifications, as our results suggest here. Specifically, TAY of PR descent who do not endorse a religious affiliation are almost twice as likely as those who identify as Catholic to have illicit SUDs and 1.5 times as likely as Non-Catholic Christian to have any SUD. From the perspective of screening, prevention, and treatment of SUDs, these findings suggest that it would behoove pediatricians, internists, family doctors, and mental health professionals, as well as researchers, to take into consideration their patients' religious identities. In addition, it would be helpful for clinicians and researchers to learn more about the role of religious community for support.

Interestingly, the results indicate that the *type* of religious affiliation—Catholic, Non-Catholic Christian, Other/Mixed—matters when it comes to assessing SUD risk. This is supported by the fact that specific religious affiliations were inversely associated with SUDs, whereas when we tested *any* religious affiliation vs. no religious affiliation (Catholic, Non-Catholic Christian, Other/Mixed vs. None), the protective effect of religious affiliation became non-significant. While this finding may surprise some—given that multiple studies have suggested that various measures of religious/spiritual identity, practice, and belief—independent of religious tradition—are associated with lower rates of SUDs (6, 16, 18), other studies have suggested that religiosity/spirituality is a risk factor for SUDs. We found that the specific religious affiliation that one's identity, practices, and beliefs is embedded in determines whether religious affiliation is protective or not, which may explain the above inconsistency in the literature. The finding is novel and interesting from a clinical perspective because it suggests that merely assessing for religious affiliation (yes or no) may be less helpful for stratifying risk as inquiring about a specific religious affiliation. In other words, to truly understand a patient's risk, a clinician might need to inquire further, and ask about one's particular religious affiliation.

Finally, while the findings related to the Other/Mixed category were non-significant, they nevertheless suggest that this group merits further study. This is because the Other/Mixed category was at an elevated risk for all SUDs in comparison to all other affiliations. Indeed, the finding was non-significant, so we cannot draw any firm conclusions about the association between Other/Mixed and SUDs, but we would argue that these findings indicate that further study in a bigger sample is indicated. If our findings are replicated in a sample with adequate power, one might conclude that the benefits of belonging to a religious tradition may not apply when one does not identify with one of the major religious traditions. However, to say one way or another, further research is necessary.

### Effects of site and gender

With respect to the interactive effects of site, our study provides evidence to suggest that the impact of endorsing no religious affiliation is greater in PR than in the SBx when it comes to risk for illicit SUDs. Further, the demographic data also show that having no religious affiliation is less common in PR than in the SBx. Together, these two findings may explain why no religious affiliation is a risk factor in PR but not the SBx: having no religious affiliation, or belonging to a religious group out of the mainstream might be more related to reduced availability of social support in PR but not the SBx. In this way, the risk for illicit SUDs that might result from not having a religious affiliation might be relative to social context, leading to greater risk in communities in which no religious affiliation is less common. This finding emphasizes the critical point that religious affiliation does not occur in a vacuum—it is a culturally-bound phenomenon that is embedded in and inseparable from community and context.

In terms of the role of gender in the association between religious affiliation and SUDs, we found no evidence of an interaction. Such a finding might lead one to conclude that gender roles and identities in Puerto Rican religious communities do not intersect to impact the use of substances among TAY. However, it is also possible that Puerto Rican gender identities can and do interact with religious affiliation to impact risk for SUDs, but they do so in complex and sometimes contradictory ways. For example, traditional Puerto Rican notions of masculinity (*machismo)*, which are embedded in and stem from a Christian religio-cultural context, may contribute to increased odds of SUDs (29). On the other hand, recent ethnographic work on male Puerto Rican evangelist street ministries suggests that Pentecostal communities promote an alternative vision of maleness—termed, “new masculinity”—that is linked to abstinence and thus lower odds of SUDs (30). Likewise among women of Puerto Rican descent, dueling notions of femininity—*marianismo* and *hembrismo*—might intersect with religious communities to impact SUD risk, as suggested by Comas-Diaz (31). In any case, research that incorporates qualitative and ethnographic methods to appreciate the complexity and nuance of these relationships is needed to clarify the complex association between gender and religion. Thus, we view this “non-finding” as a call for further investigation.

### Limitations

This research is novel and provides results that contribute to our understanding of cultural and contextual factors relevant to SUDs in Puerto Rican TAY. However, there are several limitations that are worth noting. First, while the grouping of religious identities into Catholic, Non-Catholic Christian, Other/Mixed, and None was based on the expertise of Saunders, who holds a PhD in religious studies, one might argue that the collapsing of diverse religious denominations into just four categories elides significant differences in beliefs and practices that could impact SUD risk. However, the sample size was not large enough to test for differences among all individual denominations, and the religious affiliation groups were categorized based on similarities in belief and practice among intra-group denominations.

Second, as noted above, the Other/Mixed category was small in comparison to the other three groups, and heterogeneous. Specifically, it contained persons who identified as Jewish, Muslim, and Mita Congregation, among others, as well as people who identified themselves as belonging to more than one group. These factors prohibit firm conclusions about the impact of belonging to this category.

Third, given that multiple hypotheses were tested, these findings could be the result of Type I error and thus warrant replication.

Fourth, because the associations studied were cross-sectional, reverse causation is possible, and our results may reflect the effects of low use of substances in one's choice of a religious affiliation.

Fifth, the data were collected across four years (2013–2017), and the age range of participants during data collection was 15–29. As such, it is possible that older participants whose data were collected in 2013 may experience and understand religious affiliation/non-affiliation differently than, say, younger participants whose data were collected in 2017. While we adjusted for age in our analysis, we did not adjust for year of data collection which introduces heterogeneity that we did not address. Therefore, results presented here do not account for potentially shifting attitudes toward religious affiliation in 15–29-year-olds during the period between 2013 and 2017, and instead provide a courser-grain representation of the relationship between religious affiliation and substance use-risk compared to earlier generations.

Finally, other kinds of intersecting identities, such as gender identity and sexual orientation may also influence the relationship between religious affiliation and substance use risk, particularly for individuals who feel discriminated against due to their identity by others in their religious group. However, the results presented here do not take this into account. Thus, examining interactions between gender identity, sexual orientation and religious affiliation, in their association with substance use risk is an important topic for future studies.

## Conclusion

In this paper we report on the reverse correlation between religious affiliation and substance use disorders among TAY of Puerto Rican descent raised in two social contexts, describing several notable demographic and clinical findings with implications for prevention and treatment efforts. Demographically, the number of Puerto Rican TAY who do not identify with a religious tradition is growing in comparison to prior generations. This is a clinically relevant finding because those who do not identify with a religious tradition are twice as likely as Catholics to have an illicit SUD, and 1.5 times as likely as Non-Catholic Christians to have any SUD. Interestingly, not having a religious affiliation is particularly impactful in PR, but not the SBx, in relation to risk for illicit SUD, highlighting the importance of social context in understanding the relationship between religious affiliation and SUDS. Altogether, these findings underscore the evolving and culturally embedded nature of religious affiliation (and non-affiliation), including its association with SUDs among TAY of Puerto Rican descent, and should implore clinicians working with such populations to seek to better understand their patient's religious affiliation when it comes to assessing for risk of SUDs.

## Data availability statement

The raw data supporting the conclusions of this article will be made available by the authors, without undue reservation.

## Ethics statement

The studies involving human participants were reviewed and approved by New York State Psychiatric Institute; University of Puerto Rico Medical School. Written informed consent to participate in this study was provided by the participants' legal guardian/next of kin.

## Author contributions

TC and CD had full access to all the data in the study and take full responsibility for the integrity of the data and the accuracy of the data analysis. TS, TC, and CD contributed in concept and design. DS, TS, TC, and CD drafted the manuscript. TC contributed to the statistical analysis. MA, GC, and CD obtained funding. All authors contributed to acquisition, analysis, or interpretation of data, and critical revision of the manuscript for important intellectual content.
